# Calories Purchased by Hospital Employees After Implementation of a Cafeteria Traffic Light–Labeling and Choice Architecture Program

**DOI:** 10.1001/jamanetworkopen.2019.6789

**Published:** 2019-07-10

**Authors:** Anne N. Thorndike, Emily D. Gelsomin, Jessica L. McCurley, Douglas E. Levy

**Affiliations:** 1 Division of General Internal Medicine, Department of Medicine, Massachusetts General Hospital, Boston; 2 Harvard Medical School, Boston, Massachusetts; 3 Department of Nutrition and Food Services, Massachusetts General Hospital, Boston; 4 Health Policy Research Center at the Mongan Institute, Department of Medicine, Massachusetts General Hospital, Boston

## Abstract

**Importance:**

Nearly one-third of the 150 million US adults who are employed are obese. A workplace program that reduces employees’ caloric intake could help prevent obesity.

**Objective:**

To determine if implementation of a hospital workplace cafeteria healthy eating program was associated with reduction in calories purchased by employees over 2 years.

**Design, Setting, and Participants:**

A longitudinal cohort study of 5695 employees who purchased food with their employee identification card before and after implementation of a workplace cafeteria healthy eating program was conducted from December 1, 2009, to February 29, 2012, in a hospital in Boston, Massachusetts. Statistical analysis was conducted from April 6, 2018, to May 14, 2019.

**Exposure:**

After a 3-month baseline period, “traffic light” food labels (in which green indicates healthy, yellow indicates less healthy, and red indicates least healthy) and choice architecture (product placement) changes were implemented permanently.

**Main Outcome and Measures:**

Main outcomes were changes in calories of employees’ purchases from baseline (December 1, 2009, to February 28, 2010) to the same quarter 1 year (December 1, 2010, to February 28, 2011) and 2 years (December 1, 2011, to February 29, 2012) later. Purchases were obtained from sales data linked to employee identification cards. The hypothesized association of estimated changes in daily calories consumed with employees’ weight was calculated using a dynamic model of weight change. Analyses were adjusted for age, sex, race/ethnicity, and job type.

**Results:**

Among the 5695 employees in the study, 4057 were women and 1638 were men, and the mean (SD) age was 40 (12) years. Baseline purchases had a mean of 565 kcal per transaction (95% CI, 558-572 kcal) and decreased 19 kcal per transaction (95% CI, −23 to −15 kcal) at 1 year and 35 kcal per transaction (95% CI, −39 to −31 kcal) at 2 years (−6.2%; *P* < .001) relative to baseline. The largest reduction was from red-labeled items, decreasing 42 kcal per transaction at 2 years (95% CI, −45 to −39 kcal) from a mean of 183 kcal per transaction (95% CI, 177-188 kcal) at baseline (−23.0%; *P* < .001). Calories from green-labeled items increased 6 kcal per transaction (95% CI, 3-9 kcal) from a mean of 152 kcal per transaction (95% CI, 149-155 kcal) (4.0%; *P* < .001), and calories from yellow-labeled items did not change. Among frequent purchasers (≥36 transactions per quarter; n = 453), total kilocalories per quarter at baseline was 41 784, which, averaged over the entire follow-up, decreased a mean of 4275 kcal/quarter (95% CI, −5325 to −3224 kcal) over 2 years. Assuming no compensatory changes in diet or activity, this equated to a reduction of 47 kcal per day and estimated a 2.0-kg weight loss over 3 years.

**Conclusions and Relevance:**

A workplace cafeteria traffic light–labeling and choice architecture program was associated with a sustained decrease in calories purchased, particularly from unhealthy foods. Point-of-purchase programs could help improve dietary intake and prevent obesity in employees.

## Key Points

**Question:**

Is a workplace program to promote healthy food choices using traffic light labels and choice architecture (product placement) changes associated with sustained reduction in calories purchased by employees?

**Findings:**

This cohort study of 5695 hospital employees who used the workplace cafeteria found that implementation of a traffic light–labeling and choice architecture program was associated with a 6.2% decrease in calories per transaction over 2 years, including a 23.0% decrease in calories from the least healthy foods.

**Meaning:**

Workplace point-of-purchase programs to promote healthy food choices could help improve dietary intake and prevent weight gain in employee populations.

## Teaser

This cohort study examines whether implementation of a traffic light–labeling and choice architecture program in a hospital workplace cafeteria is associated with reduction in calories purchased by employees over 2 years.

## Introduction

Nearly 150 million Americans are employed, and most spend half their waking hours at work.^1^ One-third of workers are obese, and the prevalence of obesity is increasing in all industries, including the health care industry.^2,3^ Obesity and diet-related diseases are reported to contribute to higher absenteeism and lower productivity of employees and to approximately $200 billion in US health care costs.^4,5^ Employees frequently eat meals acquired at work, and a recent nationally representative household survey found that workplace food was high in calories from saturated fat and sugars.^6^ Effective strategies for reducing nonnutritive energy intake during the workday could help address the rising prevalence of obesity in the United States and worldwide.

One strategy to promote healthier food choices by employees is implementing point-of-purchase programs in workplace cafeterias and other food retail settings.^7,8,9^ Labeling menus and menu boards with calorie counts of food items is now mandated by US federal law for chain restaurants, grocery stores, and other food retailers with 20 or more locations.^10^ More simplified labeling, such as “traffic light” labels, provide understandable information about the relative healthfulness of food and beverages and can be placed on menu boards, shelf labels, and individual packages to provide quick nutrition information to help employees make healthier choices.^11,12,13^ Choice architecture (eg, product placement) interventions make it easier and more convenient for employees to choose a healthy item without having to consider detailed nutrition information.^7,12,14^ Both strategies have been shown to promote healthier food purchases in workplace settings.^7,12,15^

Research evaluating the effectiveness of food labeling interventions for reducing calories purchased in cafeterias, restaurants, and other retail environments has been mixed but suggests that calorie labeling of food items prompts individuals to choose lower-calorie items in some settings.^9,16^ However, all the prior studies on this topic were either laboratory based or cross-sectional and typically assessed a single food or meal choice. It is unknown if labeling interventions are associated with sustained reductions in calorie intake that could help prevent weight gain and improve health or if there are only temporary effects of labeling, after which most people revert to higher-calorie choices.

Demonstrating the long-term effectiveness of workplace point-of-purchase interventions is particularly relevant for midsize and large employers who need solutions to address rising health care expenses owing to employees’ obesity-related illnesses, including diabetes and cardiovascular disease.^5^ Currently, most employers do not provide point-of-purchase programs to encourage healthier food choices. A 2013 consumer survey, including 2101 employed adults, showed that only 19.6% reported that their employer provided “opportunities to eat a healthy diet,”^17^^(p100)^ and 17.0% reported that there were “signs or labels in the cafeteria or vending area to help employees make healthy food and beverage choices.”^17^^(p100)^

Two studies previously demonstrated that a cafeteria traffic light–labeling and choice architecture program in a large hospital workplace resulted in a higher proportion of purchases of green-labeled foods (healthiest purchases) and lower proportion of purchases of red-labeled foods (least healthy purchases) over 2 years.^12,18^ The present study is a retrospective analysis, using newly available item-level calorie data, of calories purchased by a longitudinal cohort of 5695 employees who used the cafeteria at baseline (preimplementation) and during the 2-year follow-up (postimplementation) period. We examined changes in calories purchased over time, including calories from the least healthy purchases (red-labeled items) and from the healthiest purchases (green-labeled items). We hypothesized the association of the change in calorie intake with employees’ weight in a subset of employees who purchased food from the cafeteria most frequently.

## Methods

### Study Design

This is a longitudinal cohort study of employees at Massachusetts General Hospital who used the main hospital cafeteria from December 2009 through February 2012. Baseline cafeteria sales data were collected from December 1, 2009, to February 28, 2010. A traffic light–labeling intervention (in which green indicates healthy, yellow indicates less healthy, and red indicates least healthy) started March 1, 2010, and choice architecture changes were added starting June 1, 2010. Both interventions were implemented as permanent changes in the main cafeteria and subsequently expanded to all cafeterias in 2015. A previous study reported on the sales of green-, yellow-, and red-labeled food and beverages by all cafeteria customers and by hospital employees during 2 years.^12^ A database linking the calorie content of items sold in the cafeteria with sales data was created in 2016 during the startup phase of a randomized clinical trial,^19^ making the calorie-based analyses feasible for the first time. The present study is a retrospective analysis of the change in calories purchased by employees before and after implementation of the cafeteria program in 2010. This research was deemed exempt from review by the Partners Healthcare Institutional Review Board on September 28, 2009, per the regulations found at 45 CFR 46.101(b) (2) (use of educational tests, survey procedures, interview procedures, or observation of public behavior). Study participant consent was waived, and all study data were deidentified. This study followed the Strengthening the Reporting of Observational Studies in Epidemiology (STROBE) reporting guideline.

### Setting and Participants

Massachusetts General Hospital is a large academic hospital in Boston, Massachusetts, with 1 main cafeteria and 4 smaller onsite cafeterias. The cafeterias are operated by the hospital’s food and nutrition services, and no outside food vendors are located on campus. There are 9 vending machines in the entire hospital. The main cafeteria serves employees, patients, and visitors 7 days a week between 6:30 am and 8:00 pm. In 2009, Massachusetts General Hospital employed approximately 22 000 individuals with the following demographics: 77% were 35 years of age or older, 65% were female, and 28% were nonwhite. Job categories included the following: 53% clinicians or researchers, 17% clerical or office workers, 14% other professionals, 10% skilled trades, and 6% service workers. Employees have the option of paying for cafeteria purchases by direct payroll deduction using their employee identification (ID) card. We retrospectively identified a cohort of 5695 employees who used their IDs to make purchases at the main cafeteria, were continuously employed from December 2009 to February 2012, and had human resources data available. To assess the association of the healthy eating program on purchasing by employees who used the cafeteria most frequently, we also analyzed a subset of employees who made 36 purchases or more in the cafeteria during each 3-month quarter (mean, ≥3 purchases per week) continuously from baseline to the end of the 2-year follow-up.

### Cafeteria Point-of-Purchase Program

As has been described elsewhere, traffic light labeling and choice architecture changes were implemented sequentially after collecting baseline sales data for 3 months.^18^ The labeling system was originally based on the 2005 US Department of Agriculture Dietary Guidelines.^20^ Every food item was labeled as red, yellow, or green using an algorithm based on 3 positive criteria (fruit or vegetable, whole grain, and lean protein or low-fat dairy as the main ingredient) and 2 negative criteria (saturated fat and caloric content). Items with more positive than negative criteria were green, items with equal positive and negative criteria were yellow, and items with more negative than positive criteria were red. All beverages with 0 calories were green. The labels were placed on individually packaged items, menu boards, and shelf labels depending on the type of food item and packaging. Permanent and visible signs were posted throughout the cafeteria to explain the traffic light labels. Choice architecture changes were implemented 3 months after labeling to make certain green-labeled items more visible and convenient for purchase. These changes included rearranging beverage refrigerators, chip racks, and premade sandwiches to have the healthiest choices at eye level and placing baskets of bottled water near food stations. Calorie information was available to employees through manufacturers’ labels (for prepackaged goods) or in information booklets available in the cafeteria, but calories were not highlighted as part of the traffic light system. There were no significant changes in food or beverage items offered during the study period.

### Measures and Outcomes

#### Employee Demographics

Employee purchases were linked to sociodemographic data from human resources files. Data were available on age, sex, job type, and self-reported race/ethnicity (white, black, Asian, or Latino). Human resources data did not provide information on race and ethnicity separately. For research purposes, job types were aggregated into 5 categories that roughly correlated with education attainment: service workers (manual and/or unskilled laborers), support staff, technicians (eg, radiology technicians and respiratory therapists), professionals (eg, occupational therapists and pharmacists), and management and clinicians (eg, hospital managers, physicians, and nurses).

#### Cafeteria Sales

Sales data from cash registers linked to employee IDs were used to track purchases. A database of cafeteria items, including their traffic light assignment, was used for prior analyses of cafeteria purchases.^12,15,18^ Items were categorized as beverages, entrees (eg, sandwich, hot meal, or salad), or other foods (eg, snacks, desserts, hot sides, or fruit).

#### Calories

In 2016, the cafeteria sales database was updated to include calorie information. Calories for all items were manually verified by a registered dietitian (E.D.G.) using a food-service program that provided nutrient data for hospital-generated standardized recipes created by food production managers. This information was taken from nutrition resources, such as the US Department of Agriculture food composition database, as well as from vendor product information when available. Most prepackaged products had nutrition data available in the food-service program or from the nutrition facts labels. All calorie information was provided per portion sold except for items sold in bulk, which were purchased by weight. For most items sold by weight (eg, frozen yogurt), caloric content was calculated by multiplying the weight of the item (included in the sales database) by the calories per ounce. Although salad was sold by weight, the salad bar offered items with varying caloric content, including leafy greens, beans, cheeses, grains, and dressings. To estimate calories in a salad, a pilot study of 127 employees was conducted in 2015 to determine the mean number of calories per ounce.^19^ Salads were considered green unless they weighed more than 1 pound, in which case they were labeled yellow. The main measures for the current study were calories per transaction purchased by employees during the baseline quarter (December 1, 2009, to February 28, 2010) and during each quarter during the next 2 years. Other measures included the total number of calories purchased each quarter by the subset of employees who were frequent purchasers (≥36 transactions per quarter).

#### Dynamic Model of Weight Change

We used a dynamic model of weight change to hypothesize the association of the program and the change in calories with employees’ weight.^21^ The dynamic model accounts for energy expenditure adaptations that occur with weight loss. Using a US population-averaged model, a permanent change of 24 kcal per day would lead to a weight change of about 1 kg, or the equivalent of 10 kcal per day for every 0.5 kg. The dynamic model estimates that it will take 1 year to achieve half of the total weight loss, and 95% of the weight loss will occur by 3 years. This model provides a more realistic estimate of population-level intervention effects compared with the static weight loss rule, which does not account for energy adaptations over time.^22^

### Statistical Analysis

All statistical analyses were conducted from April 6, 2018, to May 14, 2019. Mean calories per transaction were calculated for all purchases during each 3-month period from baseline through the 2-year follow-up, including kilocalories per transaction from red, yellow, and green items. The mean number of kilocalories per transaction purchased during the baseline quarter (December 1, 2009, to February 28, 2010) was compared with kilocalories per transaction at 1 year (December 1, 2010, to February 28, 2011) and 2 years (December 1, 2011, to February 29, 2012) to eliminate seasonal effects. Among frequent purchasers, changes in total kilocalories per quarter from baseline to 1 and 2 years were also assessed. All analyses examined within-person changes using generalized least squares regression adjusting for age, sex, race/ethnicity, and job type and including an employee random effect. Baseline calorie levels were assessed using unadjusted data; values presented at subsequent time points were regression-adjusted changes. Data were analyzed using Stata, version 15.1 (StataCorp). All *P* values were from 2-sided tests, and the results were deemed statistically significant at *P* < .05.

To estimate the change in daily calories purchased, we calculated the mean reduction in total calories purchased per quarter (90 days) during the 2-year period and divided by 90 days to determine the mean reduction in calories per day. We first determined 1-year weight loss that would be estimated using the static model of weight change (assuming a 10-kcal/d change = 0.5-kg weight change).^21^ To determine 1-year weight change using the dynamic model, we then multiplied the number estimated by the static model by 50%, and to estimate the 3-year effect, we multiplied by 95%.^21^ For all weight change estimates, we made assumptions that employees consumed all the calories they purchased and that they had no changes in dietary intake outside of work or changes in physical activity.

## Results

### Employee Characteristics

A total of 5695 employees (4057 women and 1638 men; mean [SD] age, 40 [12] years) made cafeteria purchases during the study period, and 453 of these employees were frequent purchasers, with 36 transactions or more every quarter from December 1, 2009, to February 29, 2012. Table 1 shows the characteristics of the entire employee cohort and of the subset of frequent purchasers. Comparisons between employees who were frequent purchasers and those who were not are shown in eTable 1 in the Supplement. The overall cohort had a mean of 26 transactions per quarter compared with 76 transactions per quarter by frequent purchasers. Compared with the full cohort, the frequent purchasers were older and more likely to be male and from job types requiring a lower educational level.

**Table 1.** Characteristics of Employees Who Used the Cafeteria From December 2009 through February 2012

Characteristic	No. (%)	
	All Employees (N = 5695)	Frequent Purchasers (n = 453)^a^
Age, mean (SD), y	40 (12)	43 (12)
No. of transactions per quarter, mean (SD)	26 (25)	76 (30)
Female sex	4057 (71.2)	267 (58.9)
Race/ethnicity		
Black	568 (10.0)	63 (13.9)
Latino/Hispanic	410 (7.2)	35 (7.7)
Asian	571 (10.0)	24 (5.3)
White	4146 (72.8)	331 (73.1)
Job type^b^		
Management or clinician	2991 (52.5)	201 (44.4)
Professionals	1156 (20.3)	76 (16.8)
Technicians	481 (8.4)	62 (13.7)
Administrative support	693 (12.2)	61 (13.5)
Service workers	374 (6.6)	53 (11.7)
Full-time employment	4278 (75.1)	397 (87.6)

^a^ Frequent purchasers include employees who made a mean of at least 3 purchases per week (≥36 purchases per quarter) from December 1, 2009, to February 28, 2012.

^b^ Percentages may not total 100% because of rounding.

### Entire Employee Cohort

**Figure 1.**
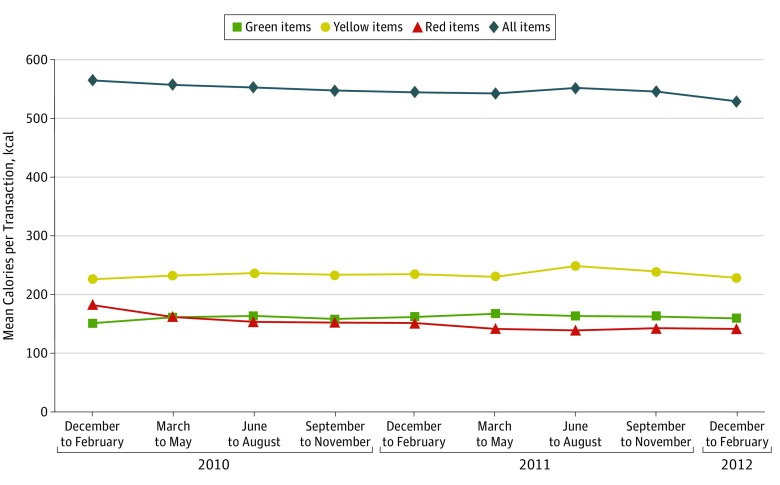
Mean Calories per Transaction for All Employees (N = 5695) Values after baseline (March 1, 2010, to February 29, 2012) are calculated using regression-adjusted differences, controlling for age, sex, race/ethnicity, job type, and full-time or part-time status as well as person and quarter random effects.

Mean calories purchased per transaction each quarter during 2 years are shown in Figure 1. At baseline (December 1, 2009, to February 28, 2010) before implementation of the program, employees’ purchases had a mean of 565 kcal per transaction (95% CI, 558-572 kcal). In regression-adjusted analyses, this amount decreased 19 kcal per transaction (95% CI, −23 to −15 kcal) at 1 year (December 1, 2010, to February 28, 2011) and 35 kcal per transaction (95% CI, −39 to −31 kcal) at 2 years (December 1, 2011, to February 29, 2012) (6.2% decrease; adjusted *P* < .001). The largest contributing factor was from a reduction in calories from red-labeled items, which decreased 42 kcal per transaction at 2 years (95% CI, −45 to −39 kcal) relative to 183 kcal per transaction (95% CI, 177-188 kcal) at baseline (23.0% decrease; adjusted *P* < .001). Calories from green-labeled items increased 6 kcal per transaction (95% CI, 3-9 kcal) from 152 kcal per transaction (95% CI, 149-155 kcal) at baseline (4.0% increase; adjusted *P* < .001), and calories from yellow-labeled items did not change significantly during the study period.

Table 2 shows the change in kilocalories per transaction at 1 and 2 years by employee demographics. At baseline, the mean number of kilocalories per transaction was higher among employees who were younger, male, nonwhite, and in job types that required a lower educational level. After adjusting for employee characteristics, the changes in calories per transaction over 2 years were significantly different by race/ethnicity, job type, and part-time job status but not by age or sex. Black, white, and Asian employees had the largest reductions in kilocalories per transaction, while Latino or Hispanic employees did not have a statistically significant reduction in kilocalories per transaction at 2 years. Part-time employees had somewhat larger reductions in kilocalories per transaction than full-time employees, but both groups decreased significantly.

**Table 2.** Change in Calories per Transaction by Characteristics of 5695 Employees

Characteristic	Baseline Kilocalories per Transaction (December 2009 to February 2010)	Change in Kilocalories per Transaction (95% CI)^a^		*P* Value for Interaction^b^
		December 2010 to February 2011	December 2011 to February 2012	
All employees	565	−19 (−23 to −15)	−35 (−39 to −31)	NA
Age category, y				
18-30	584	−28 (−36 to −21)	−40 (−49 to −32)	.07
31-40	575	−11 (−18 to −3)	−31 (−39 to −23)	
41-50	571	−22 (−30 to −13)	−43 (−51 to −34)	
>50	528	−16 (−24 to −7)	−27 (−36 to −19)	
Sex				
Male	608	−17 (−24 to −10)	−34 (−41 to −26)	.25
Female	543	−19 (−24 to −15)	−36 (−40 to −31)	
Race/ethnicity				
Black	623	−5 (−17 to 7)	−38 (−50 to −25)	<.001
Latino or Hispanic	608	3 (−11 to 17)	−19 (−33 to 4)	
Asian	534	−21 (−34 to −8)	−42 (−57 to −28)	
White	554	−23 (−27 to −18)	−36 (−40 to −31)	
Job type				
Manager or clinicians	556	−23 (−28 to −17)	−41 (−46 to −35)	.05
Professionals	529	−12 (−22 to −3)	−32 (−42 to −22)	
Technicians	596	−23 (−36 to −10)	−26 (−40 to −13)	
Administrative	594	−20 (−31 to −9)	−30 (−42 to −19)	
Service workers	607	2 (−12 to 16)	−18 (−33 to −4)	
Employment				
Full time	562	−15 (−20 to −11)	−33 (−37 to −28)	.001
Part time	575	−29 (−37 to −22)	−42 (−51 to −34)	

Abbreviation: NA, not applicable.

^a^ Regression-adjusted change from baseline, adjusting for age, sex, race/ethnicity, job type, and full-time or part-time status, as well as person and quarter random effects.

^b^ *P* values for employee characteristics assess interaction between change in kilocalories per transaction at each quarter and employee characteristic, adjusting for all covariates in the table.

### Employees Who Purchased Frequently

**Figure 2.**
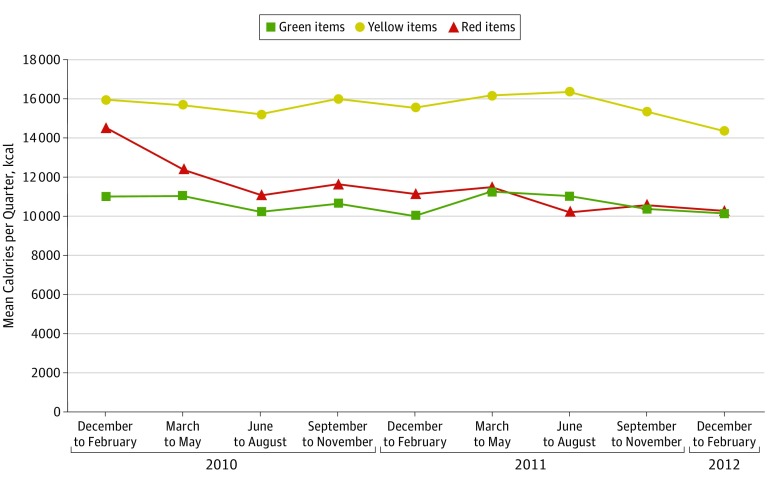
Mean Calories Purchased per Quarter by Employees Who Were Frequent Cafeteria Purchasers (n = 453) Values after baseline (March 1, 2010, to February 29, 2012) are calculated using regression-adjusted differences, controlling for age, sex, race/ethnicity, job type, and full-time or part-time status as well as person random effects.

Employees who purchased frequently purchased a mean of 41 784 kcal (95% CI, 39 982-43 585 kcal) during the baseline quarter (Table 3). Compared with the baseline quarter, this amount decreased by 4805 kcal (95% CI, −6057 to −3553 kcal) in the same quarter at 1 year and by 6756 kcal (95% CI, −8215 to −5298 kcal) in the same quarter at 2 years. The mean number of transactions decreased by 9 (95% CI, −11 to −6) and kilocalories per transaction decreased by 30 (95% CI, −42 to −19) at 2 years. Total kilocalories per quarter and kilocalories per transaction decreased for beverages, entrees, and other foods at 1 and 2 years (eFigure in the Supplement). The decrease in calories purchased at 2 years compared with baseline was 914 kcal per quarter for beverages (−22%), 3658 kcal per quarter for entrees (−17%), and −2178 kcal per quarter for other food (−14%) (adjusted *P* < .001 for all comparisons). Figure 2 shows that the largest reduction in calories at 2 years was from red-labeled items, decreasing 4196 kcal per quarter (95% CI, −5065 to −3327 kcal) from 14 498 kcal per quarter at baseline (adjusted *P* < .001). Yellow-labeled foods’ kilocalories per quarter decreased 1575 (95% CI, −2429 to −720 kcal) over 2 years from 15 913 (adjusted *P* < .001), and green-labeled foods’ kilocalories per quarter decreased 856 (95% CI, −1476 to −236 kcal) from 10 972 (adjusted *P* = .007). Sensitivity analyses revealed that effect sizes were lower when the sample was expanded to employees with 12 or more purchases per quarter or to employees with 36 or more purchases per quarter, excluding those with more than 65 purchases per quarter (eTable 2 and eTable 3 in the Supplement).

**Table 3.** Change in Transactions and Calories per Quarter by Employees Who Were Frequent Purchasers^a^

Characteristic of Transactions per Quarter	Baseline, Mean (SD), December 2009 to February 2010	1-y Change From Baseline (95% CI), December 2010 to February 2011^b^	2-y Change From Baseline (95% CI), December 2011 to February 2012^b^
All purchases			
Total kilocalories	41 784 (19 148)	−4805 (−6057 to −3553)	−6756 (−8215 to −5298)
No. of transactions	76 (30)	−7 (−9 to −5)	−9 (−11 to −6)
Kilocalories per transaction	559 (182)	−16 (−26 to −6)	−30 (−42 to −19)
Beverage purchases			
Total kilocalories	4231 (4691)	−655 (−921 to −388)	−914 (−1247 to −582)
No. of transactions	48 (31)	−3 (−5 to 1)	−3 (−5 to 0)
Kilocalories per transaction	92 (72)	−13 (−18 to −7)	−16 (−22 to −11)
Entree purchases			
Total kilocalories	22 050 (9758)	−2118 (−2831 to −1405)	−3658 (−4484 to −2832)
No. of transactions	47 (18)	−3 (−5 to −2)	−6 (−8 to −5)
Kilocalories per transaction	465 (100)	−16 (−23 to −8)	−20 (−28 to −12)
Other food purchases			
Total kilocalories	15 502 (10 577)	−2038 (−2725 to −1351)	−2178 (−2983 to −1373)
No. of transactions	47 (22)	−4 (−5 to −2)	−5 (−7 to −3)
Kilocalories per transaction	314 (112)	−15 (−22 to −8)	−11 (−20 to −2)

^a^ There were 453 frequent purchasers, with 36 or more transactions per quarter.

^b^ Regression-adjusted change from baseline, adjusting for age, sex, race/ethnicity, job type, and full-time/part-time status, as well as person random effects.

### Hypothesized Association of the Cafeteria Program With Employees’ Weight

For the dynamic model of weight change, we calculated the mean change in kilocalories per quarter for all quarters during the 2-year follow-up period (4275 kcal per quarter [95% CI, −5325 to −3224 kcal]) to estimate employees’ daily calorie reduction of 47 kcal per day. The dynamic model estimated that employees who used the cafeteria frequently would lose 1.1 kg at 1 year and 2.0 kg at 3 years after implementation of the cafeteria point-of-purchase program, assuming no other changes in dietary intake or physical activity.

## Discussion

Hospital employees purchased fewer calories at work over 2 years after implementation of a healthy eating program in the cafeteria. To our knowledge, this is the first study to evaluate longitudinal, objective food purchasing data to determine if a simple food labeling and choice architecture intervention was associated with sustained reductions in the calories of food purchased. In a subset of employees who visited the cafeteria most frequently, the estimated daily reduction in calories estimated a weight loss of up to 2 kg over 3 years. In the future, more widespread implementation of simple, healthy eating interventions in workplaces could help improve dietary intake and prevent weight gain among employees in the United States and worldwide.

Among all employees, the largest reduction in calories purchased occurred for the least healthy items in the red-labeled category, including foods high in unhealthy fat and sugar, and was sustained over 2 years. Improving diet quality, even without weight change, has been shown to reduce the risk of chronic disease.^23^ For employees who used the cafeteria most frequently, the caloric changes were associated with weight loss. Although this association was limited by the assumption that employees had no compensatory changes in diet or activity, it is reasonable to assume that the reduction in workplace calories may have at a minimum slowed the annual weight gain typically experienced by most adults.^24,25^ In a recent study of 602 hospital employees, the healthfulness of workplace cafeteria purchases was associated with overall dietary quality and obesity.^26^ However, the finding from our study that Latino employees did not have as large a reduction in calories as other racial/ethnic groups suggests that the cafeteria program was not effective for this group and warrants future study.

Previous studies evaluating the effectiveness of calorie labels for reducing calorie intake have been variable and have only analyzed cross-sectional or short-term data from receipts or self-reported purchases.^9^ Numeric calorie labels do not provide information about the relative healthfulness or nutritional quality of food. Therefore, a person might opt for a lower-calorie, small-portion entree high in unhealthy ingredients, such as saturated fat, rather than a larger-portion entree with healthier ingredients, such as whole grains. Experimental studies have demonstrated that traffic light labels improve the effectiveness of calorie labels and front-of-package nutrition labels in promoting healthier food choices.^11,13^

Effective point-of-purchase interventions that sustain healthy food choices have the potential to improve health.^27,28^ Although obesity has been increasing across all employment industry categories, an analysis of the US National Health Interview Survey found that the health care industry had the highest age-standardized prevalence of obesity.^2^ Hospitals in the United States employ nearly 5.9 million people and treat more than 750 million people a year in emergency departments, outpatient visits, and surgery departments.^29^ Therefore, millions of people are exposed to food offered at hospitals, and healthy eating programs could influence not only employees but also patients and visitors.

Large employers, especially hospitals, are responsible for purchasing large quantities of food and can be important drivers of the food industry.^8^ A recent meta-analysis evaluated the influence of food labeling on food industry practices and found evidence that food labeling resulted in industry reformulation of products to reduce trans fats and sodium.^28^ If food labeling and other strategies were implemented broadly by large employers, these changes could lead employees to increase demand for healthier food products at work and drive employers’ purchasing patterns.

### Strengths and Limitations

The strength of this study is the use of objective food purchasing data from a large longitudinal cohort of employees during a 2-year follow-up period. There are also some limitations. First, this study was a longitudinal study that did not have a control site. However, analyses of employees were based on within-person changes in purchasing and were not vulnerable to the biases associated with changing populations. In addition, purchasing patterns for all analyses showed sustained changes during the 2-year follow-up. Second, it is unknown if employees consumed all items they purchased. All cafeteria items were sold as single-serve, ready-to-eat items. Most employees were familiar with typical serving sizes, making it unlikely that they would have consistently paid for items they were not going to consume fully. Third, the number of cafeteria transactions by frequent purchasers decreased, which contributed to the reduction in calories purchased per quarter. We were unable to determine if this reduction occurred because employees consumed less food at work or because they acquired food elsewhere (eg, purchasing elsewhere or bringing from home). However, the frequent purchasers also had reductions in calories per transaction, indicating that they purchased fewer calories each time they made purchases. Fourth, this study took place in 1 large urban hospital, and results may not be generalized to other smaller or nonhospital workplaces.

## Conclusions

A traffic light–labeling and choice architecture program in a real-world hospital cafeteria setting was associated with reduction in calories purchased by employees over 2 years. More importantly, most of the decrease in calories was attributed to fewer calories from unhealthy food and beverages. Results of this study suggest that employees who used the workplace cafeteria most frequently could have lost weight or, more conservatively, avoided weight gain over 3 years. Our findings support broader implementation of healthy eating interventions in other large workplaces, particularly hospitals and health care settings, to address the unprecedented levels of obesity and obesity-related chronic diseases in the United States and worldwide.

## Notes

Supplement. **eTable 1.** Characteristics of Employees Who Used the Cafeteria from December 2009 Through February 2012: Comparison of Frequent and Nonfrequent Purchasers **eTable 2.** Change in Transactions and Calories per Quarter by Employees Who Were Moderately Frequent Purchasers (≥12 Transactions/Quarter) (N = 1916) **eTable 3.** Change in Transactions and Calories per Quarter by Employees Who Were Frequent Purchasers (≥36 Transactions/Quarter), Excluding the Most Frequent Purchasers (Those With >65 Transactions/Quarter) (N = 363) **eFigure.** Mean Calories per Quarter From Beverages, Entrees, and Other Food Items by Employees Who Were Frequent Cafeteria Purchasers (N = 453)
