# Supplementary material for: Calories Purchased by Hospital Employees After Implementation of a Cafeteria Traffic Light–Labeling and Choice Architecture Program
Source: JAMA Netw Open. 2019 Jul 10;2(7):e196789. doi: 10.1001/jamanetworkopen.2019.6789 (PMC6624805; doi:10.1001/jamanetworkopen.2019.6789)
Supplement: Supplement. — eTable 1. Characteristics of Employees Who Used the Cafeteria from December 2009 Through February 2012: Comparison of Frequent and Nonfrequent Purchasers eTable 2. Change in Transactions and Calories per Quarter by Employees Who Were Moderately Frequent Purchasers (≥12 Transactions/Quarter) (N = 1916) eTable 3. Change in Transactions and Calories per Quarter by Employees Who Were Frequent Purchasers (≥36 Transactions/Quarter), Excluding the Most Frequent Purchasers (Those With >65 Transactions/Quarter) (N = 363) eFigure. Mean Calories per Quarter From Beverages, Entrees, and Other Food Items by Employees Who Were Frequent Cafeteria Purchasers (N = 453) [file jamanetwopen-2-e196789-s001.pdf]

Supplementary Online Content

Thorndike AN, Gelsomin ED, McCurley JL, Levy DE. Calories purchased by hospital employees after implementation of a cafeteria traffic light–labeling and choice architecture program. *JAMA Netw Open.* 2019;2(7):e196789. doi:10.1001/jamanetworkopen.2019.6789

eTable 1. Characteristics of Employees Who Used the Cafeteria from December 2009 Through February 2012: Comparison of Frequent and Nonfrequent Purchasers
eTable 2. Change in Transactions and Calories per Quarter by Employees Who Were Moderately Frequent Purchasers (≥12 Transactions/Quarter) (N=1916)
eTable 3. Change in Transactions and Calories per Quarter by Employees Who Were Frequent Purchasers (≥36 Transactions/Quarter), Excluding the Most Frequent Purchasers (Those With >65 Transactions/Quarter) (N=363)
eFigure. Mean Calories per Quarter From Beverages, Entrees, and Other Food Items by Employees Who Were Frequent Cafeteria Purchasers (N=453)

This supplementary material has been provided by the authors to give readers additional information about their work.

© 2019 Thorndike AN et al. *JAMA Network Open.*

eTable 1. Characteristics of Employees Who Used the Cafeteria from December 2009 Through February 2012: Comparison of Frequent and Nonfrequent Purchasers

	**Frequent purchasers[a]** (N=453)	**All other employees** (N= 5,242)	**P-value**
Age, mean (SD)	43 (12)	40 (12)	<0.001
No. transactions per quarter, mean (SD)	76 (30)	24 (22)	<0.001
Female gender, N (%)	267 (59)	3,790 (72)	<0.001
Race/ethnicity, N (%)			<0.001
Black	63 (14)	505 (10)	
Latino/Hispanic	35 (8)	375 (7)	
Asian	24 (5)	547 (10)	
White	331 (73)	3,815 (73)	
Job type, N (%)[b]			<0.001
Management/clinician	201 (44)	2,790 (53)	
Professionals	76 (17)	1,080 (21)	
Technicians	62 (14)	419 (8)	
Administrative support	61 (14)	632 (12)	
Service workers	53 (12)	321 (6)	
Full-time employment, N (%)	397 (88)	3,881 (74)	<0.001

[a]Frequent purchasers include employees who made an average of 3 or more purchases per week (≥36 purchases per quarter) from December 1, 2009, to February 28, 2012.

[b]Percentages may not add to 100% due to rounding.

© 2019 Thorndike AN et al. *JAMA Network Open.*

eTable 2. Change in Transactions and Calories per Quarter by Employees Who Were Moderately Frequent Purchasers (≥12 Transactions/Quarter) (N=1916)

**Kcal and no. transactions per quarter**	**Baseline,** mean (SD) (Dec-Feb 2010)	**1-year change from baseline** (95% CI)[a] (Dec-Feb 2011)	**2-year change from baseline** (95% CI)[a] (Dec-Feb 2012)
All purchases			
Total kcal	27,565 (16,869)	-3,799 (-4,350, -3,248)	-5345 (-5,991, -4,699)
No. transactions	49 (18)	-5 (-6, -4)	-7 (-8, -6)
Kcal/transaction	570 (175)	-18 (-23, -13)	-32 (-39, -26)
Beverage purchases			
Total kcal	2,403 (3,345)	-489 (-594, -385)	-643 (-763, -523)
No. transactions	29 (25)	-2 (-3, -1)	-3 (-4, -2)
Kcal/transaction	86 (69)	-12 (-14, -9)	-16 (-19, -13)
Entree purchases			
Total kcal	15,680 (8,926)	-2,121 (-2,348, -1,694)	-3,075 (-3,449, -2,700)
No. transactions	33 (17)	-3 (-4, -3)	-6 (-6, -5)
Kcal/transaction	474 (103)	-16 (-20, -12)	-14 (-18, -9)
Other food purchases			
Total kcal	9,489 (8,148)	-1,317 (-1,581, -1,051)	-1,654 (-1,973, -1,335)
No. transactions	31(20)	-3 (-4, -2)	-5 (-5, -4)
Kcal/transaction	298 (115)	-11 (-15, -6)	-10 (-15, -4)

Kcal: kilocalories

[a] Regression-adjusted change from baseline, adjusting for age, sex, race/ethnicity, job type, and full-/part-time status, as well as person random effects.

© 2019 Thorndike AN et al. *JAMA Network Open.*

eTable 3. Change in Transactions and Calories per Quarter by Employees Who Were Frequent Purchasers (≥36 Transactions/Quarter), Excluding the Most Frequent Purchasers (Those With >65 Transactions/Quarter) (N=363)

**Kcal and no. transactions per quarter**	**Baseline,** mean (SD) (Dec-Feb 2010)	**1-year change from baseline** (95% CI)[a] (Dec-Feb 2011)	**2-year change from baseline** (95% CI)[a] (Dec-Feb 2012)
All purchases			
Total kcal	37,835 (16,035)	-3,706 (-5,090, -2,323)	-5,520 (-7,082, -3,957)
No. transactions	68 (21)	-5 (-7, -3)	-7 (-10, -4)
Kcal/transaction	564 (178)	-14 (-25, -3)	-29 (-42, -16)
Beverage purchases			
Total kcal	3,587 (3,900)	-552 (-844, -260)	-761 (-1,131, -391)
No. transactions	41 (23)	-2 (-3, 0)	-1 (-4, 1)
Kcal/transaction	91 (72)	-13 (-20, -7)	-17 (-24, -11)
Entree purchases			
Total kcal	20,560 (8,713)	-1,843 (-2,621, -1,065)	-3,258 (-4,118, -2,398)
No. transactions	44 (16)	-3 (-4, -1)	-6 (-7, -4)
Kcal/transaction	463 (102)	-17 (-35, -9)	-19 (-28, -10)
Other food purchases			
Total kcal	13,689 (8,717)	-1,331 (-1,907, -362)	-1,507 (-2,360, -654)
No. transactions	43 (19)	-3 (-5, -1)	-4 (-6, -2)
Kcal/transaction	308 (109)	-13 (-21, -5)	-9 (-19, 2)

Kcal: kilocalories

[a] Regression-adjusted change from baseline, adjusting for age, sex, race/ethnicity, job type, and full-/part-time status, as well as person random effects.

© 2019 Thorndike AN et al. *JAMA Network Open.*

eFigure. Mean calories per quarter from beverages, entrees, and other food items by employees who were frequent cafeteria purchasers (N=453)

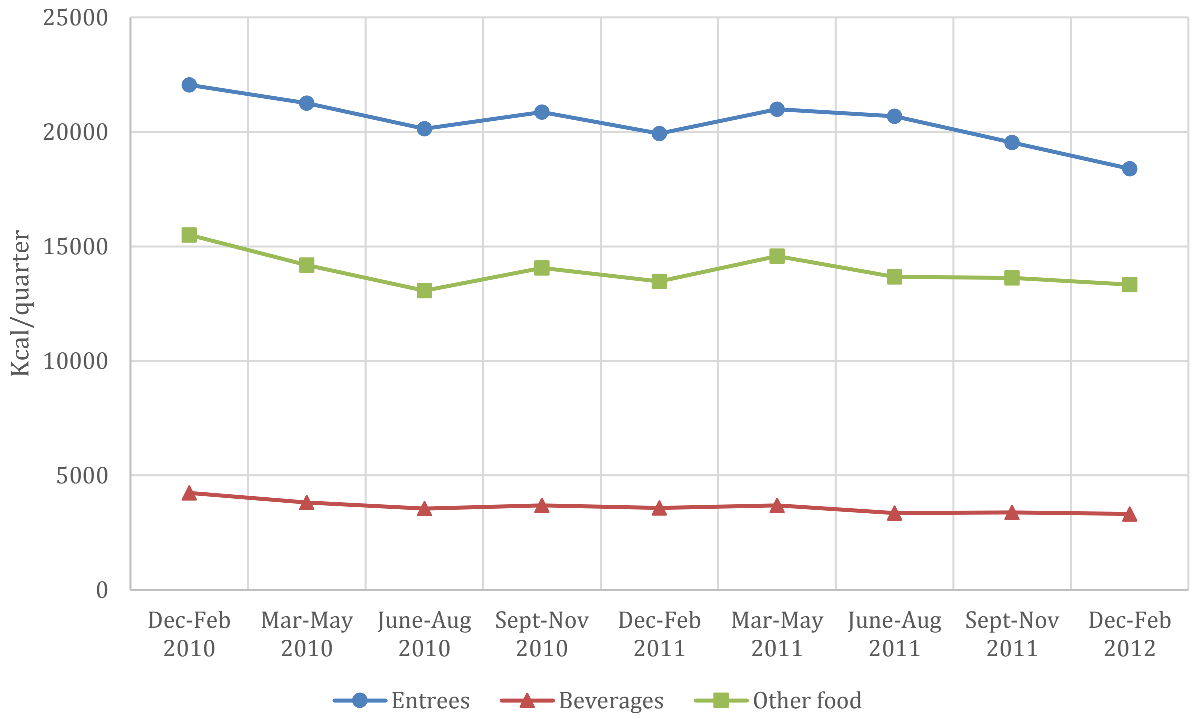

© 2019 Thorndike AN et al. *JAMA Network Open.*
